# The ATP-dependent RNA helicase HrpB plays an important role in motility and biofilm formation in *Xanthomonas citri* subsp. *citri*

**DOI:** 10.1186/s12866-016-0655-1

**Published:** 2016-03-23

**Authors:** Laís Moreira Granato, Simone Cristina Picchi, Maxuel de Oliveira Andrade, Marco Aurélio Takita, Alessandra Alves de Souza, Nian Wang, Marcos Antonio Machado

**Affiliations:** Centro de Citricultura Sylvio Moreira/IAC, Rodovia Anhanguera Km 158, Cordeirópolis, SP 13490-970 Brazil; Universidade Estadual de Campinas/UNICAMP, Instituto de Biologia, P.O. Box 6010, Campinas, SP 13083-970 Brazil; Citrus Research and Educational Center, Department of Microbiology and Cell Science, University of Florida, IFAS, 700 Experiment Station Road, Lake Alfred, FL 33850 USA

**Keywords:** RNA helicase, *Xanthomonas citri*, Biofilm, Citrus canker, Type IV pili

## Abstract

**Background:**

RNA helicases are enzymes that catalyze the separation of double-stranded RNA (dsRNA) using the free energy of ATP binding and hydrolysis. DEAD/DEAH families participate in many different aspects of RNA metabolism, including RNA synthesis, RNA folding, RNA-RNA interactions, RNA localization and RNA degradation. Several important bacterial DEAD/DEAH-box RNA helicases have been extensively studied. In this study, we characterize the ATP-dependent RNA helicase encoded by the *hrpB* (XAC0293) gene using deletion and genetic complementation assays. We provide insights into the function of the *hrpB* gene in *Xanthomonas citri* subsp. *citri* by investigating the roles of *hrpB* in biofilm formation on abiotic surfaces and host leaves, cell motility, host virulence of the citrus canker bacterium and growth *in planta*.

**Results:**

The *hrpB* gene is highly conserved in the sequenced strains of *Xanthomonas*. Mutation of the *hrpB* gene (*∆hrpB*) resulted in a significant reduction in biofilms on abiotic surfaces and host leaves. *∆hrpB* also exhibited increased cell dispersion on solid medium plates. *∆hrpB* showed reduced adhesion on biotic and abiotic surfaces and delayed development in disease symptoms when sprayed on susceptible citrus leaves. Quantitative reverse transcription-PCR assays indicated that deletion of *hrpB* reduced the expression of four type IV pili genes. The transcriptional start site of *fimA* (XAC3241) was determined using rapid amplification of 5′-cDNA Ends (5′RACE). Based on the results of *fimA* mRNA structure predictions, the *fimA* 5′ UTR may contain three different loops. HrpB may be involved in alterations to the structure of *fimA* mRNA that promote the stability of *fimA* RNA.

**Conclusions:**

Our data show that *hrpB* is involved in adherence of *Xanthomonas citri* subsp. *citri* to different surfaces. In addition, to the best of our knowledge, this is the first time that a DEAH RNA helicase has been implicated in the regulation of type IV pili in *Xanthomonas*.

**Electronic supplementary material:**

The online version of this article (doi:10.1186/s12866-016-0655-1) contains supplementary material, which is available to authorized users.

## Background

RNA helicases are enzymes that catalyze the ATP-dependent separation of double-stranded RNA (dsRNA). RNA helicases are found in all kingdoms of life [1, 2]. DEAD-box proteins, which are named for their highly conserved motif I residues (Asp-Glu-Ala-Asp), and the related DEAH, DExH and DExD families, which are commonly referred to collectively as the DExD/H helicase family, share eight conserved motifs [3–5] that have been shown to be involved in the activities and regulation of ATPases and helicases [6].

DExD/H proteins participate in many different parts of RNA metabolism, including RNA synthesis, RNA folding, RNA-RNA interactions, RNA localization and RNA degradation [6–9]. Several bacterial DEAD/DEAH-box proteins have been characterized and found to be involved in different phenotypes. For example, in *Bacillus subtilis*, two cold-induced putative RNA-helicases, CshA and CshB, are thought to be essential for cold adaption, during which they work in conjunction with cold-shock proteins to rescue misfolded mRNA molecules and to maintain proper initiation of translation at low temperatures [10]. Mutation of the *cshA* DEAD-box gene in *Staphylococcus aureus* resulted in the dysregulation of biofilm formation and hemolysis via modulation of *agr* mRNA stability [6]. Furthermore, the putative DEAD-box helicase AggH is important during auto-aggregation [11] in *Lactobacillus reuteri*. In *Listeria monocytogenes*, four putative DEAD-box RNA helicases (lmo0866, lmo1246, lmo1450 and lmo1722) are required for growth and motility [12].

HrpA, a DEAH-box RNA helicase in *Escherichia coli*, is involved in processing *daa* mRNA from a fimbrial operon. This processing event results in a stable mRNA and the up-regulation of *daa* expression relative to the levels of other proteins that are encoded by the polycistronic transcript [13]. The HrpA protein also appears to be involved in physical interactions with a variety of ribosomal proteins in *E. coli* either directly or indirectly through RNA interactions [14, 15], consistent with a possible translational-level regulatory role [8].

In our previous study, we screened *Xanthomonas citri* subsp. *citri* (*X. citri*) for mutants that were associated with effects on biofilm and identified *hrpB* (XAC0293), which encodes a probable DEAH-box RNA helicase. The function of RNA helicases in the Gram negative bacteria *X. citri* has not been explored. *X. citri* causes citrus canker, one of the most economically damaging diseases that affects citrus [16, 17]. It is spread by wind-blown rain and invades the host directly through natural openings, such as stomata, and through wounds [16]. Previous studies have shown that *X. citri* forms biofilms on leaf surface [18–21], which increases the epiphytic survival of the bacteria and plays an important role in the invasion of host intercellular spaces by *X. citri* [18, 22].

Biofilms are communities of bacterial cells that are embedded in a matrix of extracellular polymeric compounds that are attached to a surface [23]. Biofilm formation is a dynamic and complex process that generally includes the initial attachment of cells to the surface at the substratum, physiological changes within the organism, multiplication of the cells to form microcolonies and the maturation of the biofilm [21]. The stable adhesion of the bacteria to the surface is a key step in biofilm formation, and type IV pili (T4P) genes are thought to be important for cell-to-cell aggregation and adherence to surfaces [24]. The *X. citri* strain 306 contains a functional T4P [25]. The FimA proteins (XAC3241 and XAC3240), which form the major pilin subunit [26], are produced through the secretion and polymerization of pilin subunits via a process that depends on PilB, a hexameric ATPase that is associated with the bacterial inner membrane [27]. Pilus retraction is powered by another ATPase, PilT/PilU [28].

In this study, we showed that the *hrpB* (XAC0293) gene plays an important role in adherence and biofilm development in *X. citri* and that its deletion reduced the expression of type IV pili genes. Our study sheds light on the involvement of DEAH-box proteins in adhesion, biofilm formation and pathogenicity in plant-pathogen bacteria.

## Results

### *XAC0293*/*hrpB* encodes a putative ATP-dependent RNA helicase that is involved in RNA metabolism

**Fig. 1:**
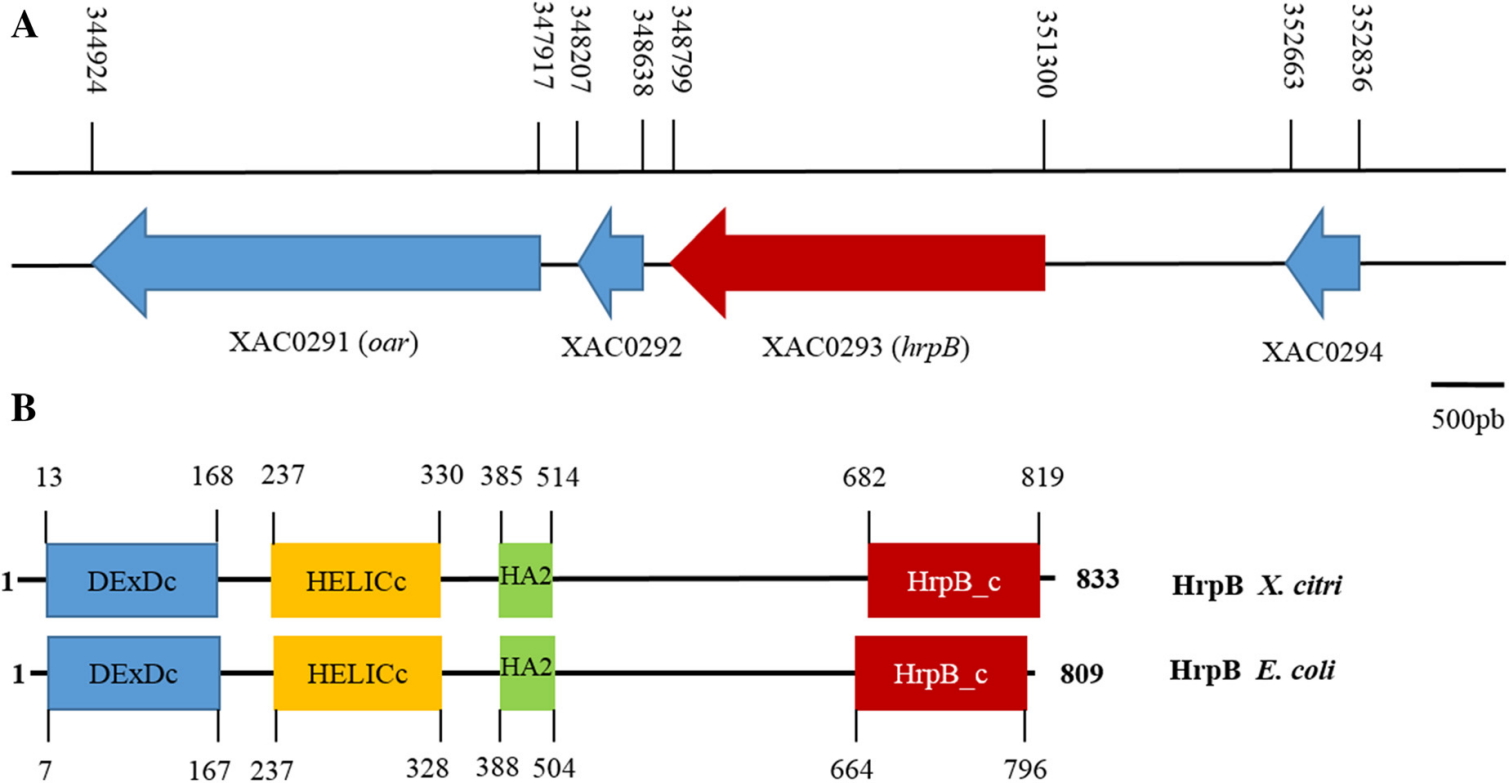
**a** Schematic diagram of the *hrpB* (XAC0293) gene in the *X. citri* subsp. *citri* strain 306 genome and the lengths of the open reading frames [29]. **b** Domain structure analyses of *hrp* *B* in *Xanthomonas citri* subsp. *citri* and *E. coli*. The domain structure predictions were performed using the Pfam protein families database. Domain symbols: DExDc, DEAD-like helicase superfamily; HELICc, Helicase superfamily c-terminal domain; HA2, Helicase-associated domain; and HrpB_c, ATP-dependent helicase C-terminal domain

**Fig. 2:**
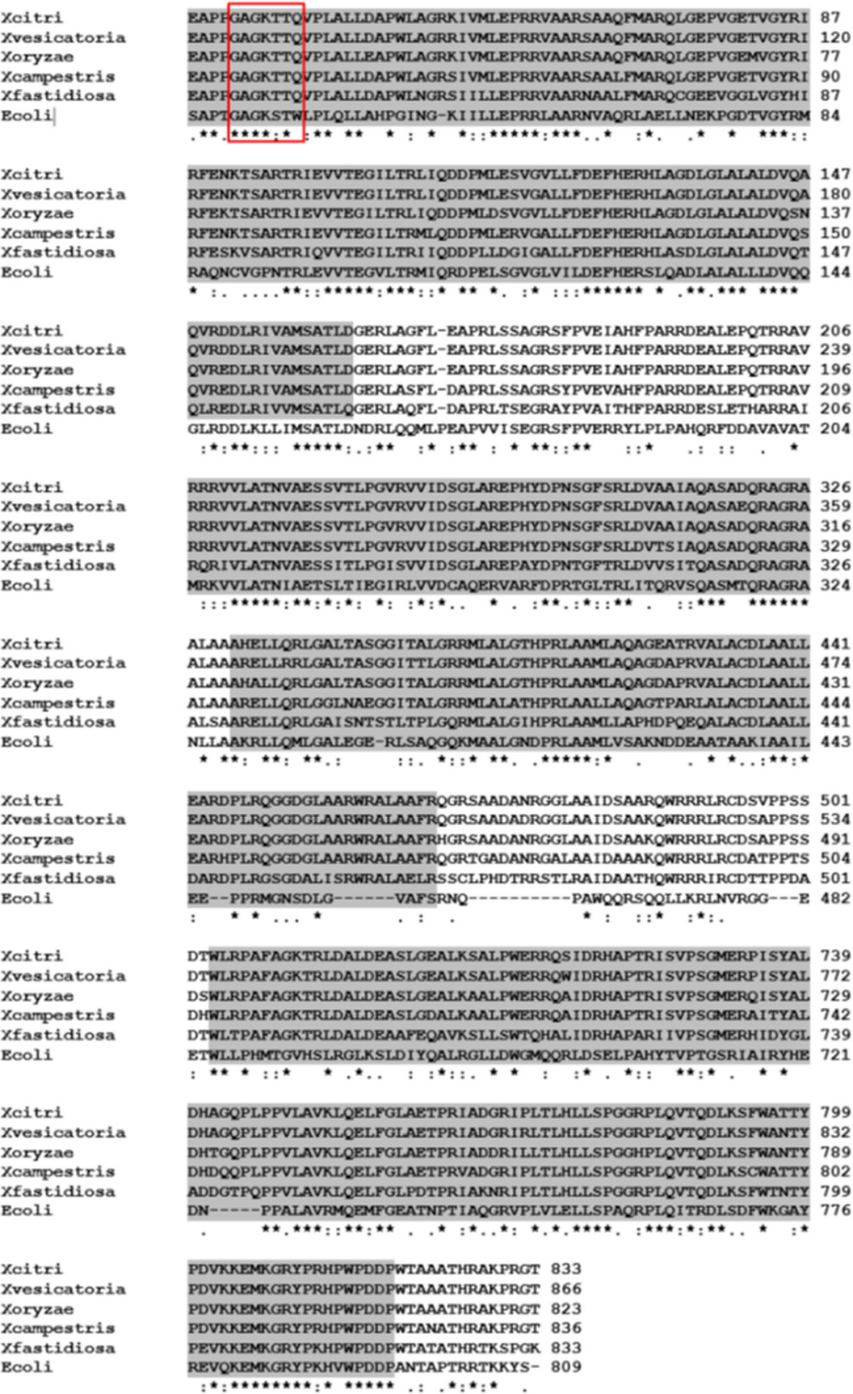
Sequence alignments of HrpB homologs. The * indicates the presence of a conserved motif. The ATP-binding region (GAGKT) that characterizes the DEAD-like helicase superfamily is indicated by a box. Abbreviations are as follows: ECHrpB, *Escherichia coli* str. K-12 str. DH10B; Xf1229, *Xylella fastidiosa* 9a5c; Xcc0275, *Xanthomonas campestris* pv. *campestris* str. ATCC 33913; Xoo4533, *Xanthomonas oryzae* pv. *oryzae* KACC 10331; Xac0293, *Xanthomonas citri* subsp. *citri*; and Xcv0300, *Xanthomonas campestris* pv. *vesicatoria* str. 85–10

The XAC0293 open reading frame (ORF) is 2501 bp in length and is located within the genome at position 348799–351300 (Fig. 1). The adjacent genes upstream (XAC0294) and downstream (XAC0292) of this location are in the same orientation and encode hypothetical proteins. XAC0293 was annotated as an 833 amino acid-long ATP-dependent RNA helicase, and the predicted pI and molecular weight (MW) of this amino acid are 9.49 and 90.9 kD (http://web.expasy.org/compute_pi/), respectively. The protein XAC0293 shares 42 % identity with the *Escherichia coli* RNA helicase HrpB, the function of which has not been described. A domain structure analysis performed using the Pfam database showed that XAC0293 contains four domains that are associated with DEAD/H-box RNA helicase proteins, including a DEAD-like helicase superfamily domain (DEXDc) at the N-terminal, a helicase superfamily c-terminal domain (HELICc), a helicase-associated domain (HA2), and an ATP-dependent helicase C-terminal domain (HrpB_c) at its C-terminal. The HrpB protein also contains two predicted ATP-binding sites, one Mg^++^-binding site and one nucleotide-binding region. Protein BLAST showed that XAC0293 is highly conserved in other *Xanthomonas* species (Fig. 2), including *X. campestris* pv. *vesicatoria* str. 85–10 (97 % identity), *X. oryzae* pv. *oryzae* KACC 10331 (95 % identity), *X. campestris* pv. *campestris* str. ATCC 33913 (91 % identity), and *Xylella fastidiosa* (72 % identity) (Table 1). In addition, a total of four ATP-dependent RNA helicases were identified in *X. citri*, including Xac0293, Xac3122, Xac2390, and Xac0442. Among these, both Xac0293 and Xac0442 are DEAD-box helicases.

**Table 1:** Protein alignments of *Xanthomonas citri* subsp. *citri* in different bacteria

Strains	Proteins	XAC0293 (*hrpB*)	
		E- value	Max ident
*Xanthomonas campestris* pv. *vesicatoria* str. 85–10	ATP-dependent helicase HrpB	0.0	97 %
*Xanthomonas oryzae* pv. *oryzae* KACC 10331	ATP-dependent RNA helicase	0.0	95 %
*Xanthomonas campestris* pv. *campestris* str. ATCC 33913	ATP-dependent RNA helicase	0.0	91 %
*Xylella fastidiosa* 9a5c	ATP-dependent helicase	0.0	72 %
*Escherichia coli* str. K-12 substr. DH10B	ATP-dependent helicase HrpB	0.0	42 %

### Mutation of *hrpB* affected biofilm formation on abiotic surfaces and host leaves in *X. citri*

**Fig. 3:**
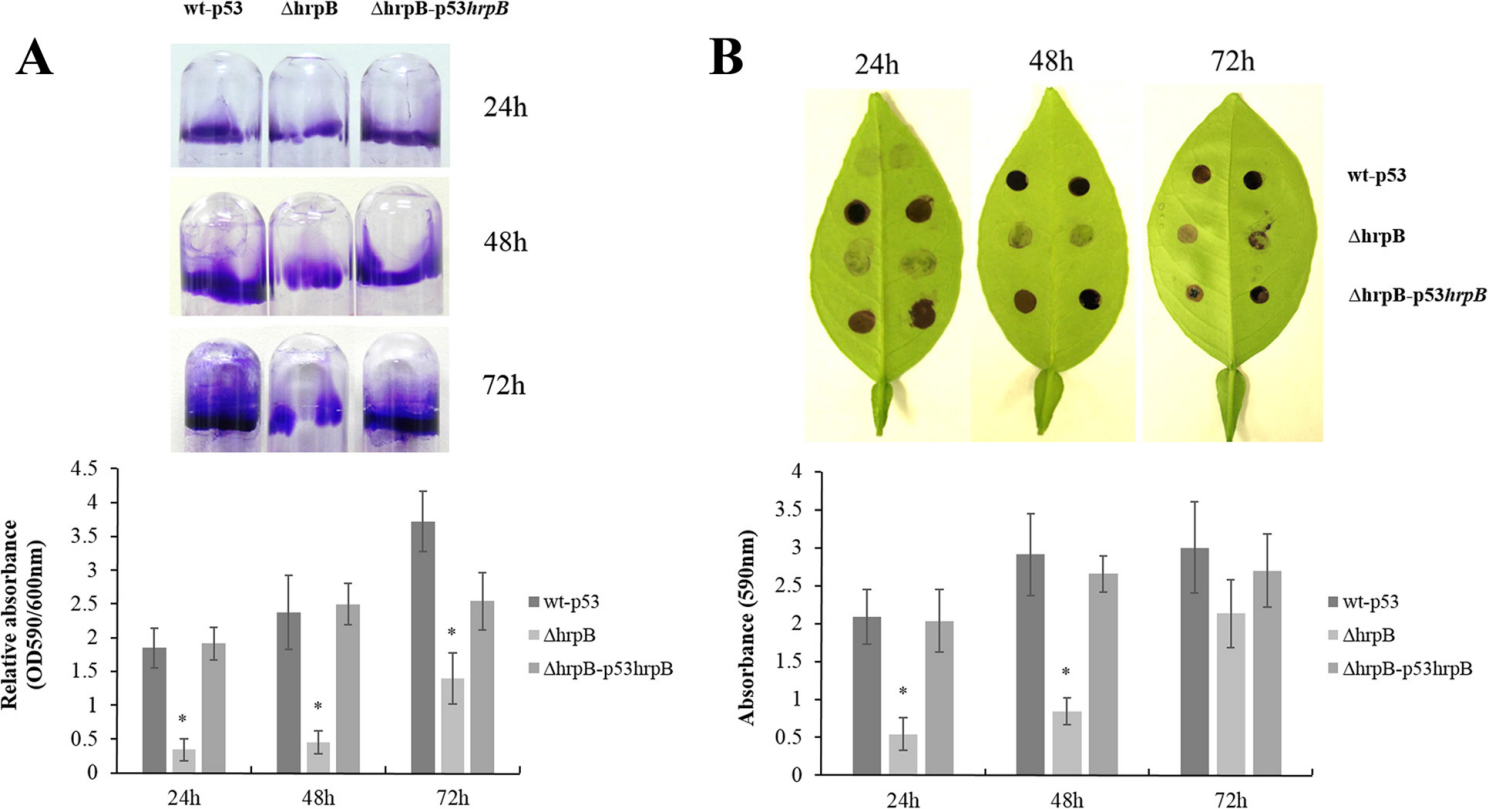
Biofilm formation by *X. citri* subsp. *citri* strain 306 and its derivatives. **a** Biofilm formation on abiotic surfaces. **b** Biofilm formation on citrus abaxial leaf surfaces. The results of the biofilm formation assays were quantified by measuring the optical density after dissolution in ethanol-acetone (70:30, v/v). Values are expressed as the means ± standard deviation. * indicates significantly different p-values (p ≤ 0.05) according to Tukey’s tests

To study the function of *hrpB*, we used an allelic exchange protocol to produce an *hrpB* mutant (∆*hrpB*) of *X. citri* strain 306, which was confirmed using PCR. The growth curve of the mutant was not different from that in the wild-type (data not shown). Biofilm development was examined in polystyrene plates and glass tubes and on sweet orange leaves. A significant reduction in biofilm formation was observed in ∆*hrpB* after 48 h of growth in NB medium containing 1 % glucose compared to the wild-type and complemented strains (Fig. 3a). Crystal violet staining was over 5 times more intense in the *X. citri* wild-type strain than in the ∆*hrpB* strain. Similar results were observed in attachment to abiotic and leaf surfaces. The ∆*hrpB* strain formed over 4 times less biofilm on host leaves (OD590 = 0.54 ± 0.20) than the wild-type strain (OD590 = 2.19 ± 0.45), and the complemented strain, ∆*hrpB*-p53*hrpB*, formed levels similar to the wild-type strain (Fig. 3b). No difference was observed in xanthan gum production between the wild-type and mutant *hrpB* strains under the tested conditions (data not shown). These findings suggest that the *hrpB* gene is involved in cell adhesion and, consequently, biofilm formation in *X. citri*.

### Deletion of *XAC0293*/*hrpB* resulted in increased motility

**Fig. 4:**
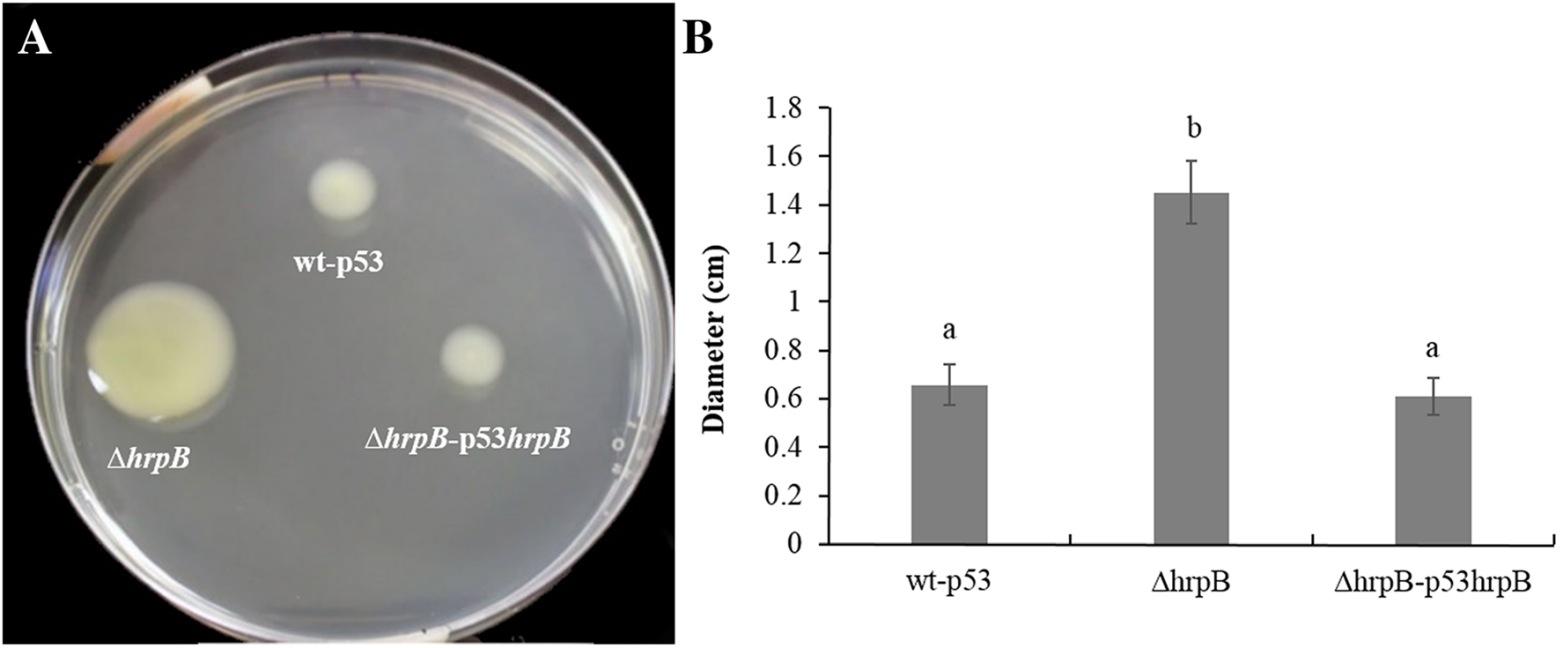
Sliding motility in *X. citri* subsp. *citri* strains. **a** ∆*hrpB* showed an increase in motility that could be restored to wild-type levels by the introduction of a plasmid containing the intact *hrpB* gene. **b** Measurements of the diameters of cell spread on plates. Cells were inoculated from bacterial cultures at exponential stage onto SB plates that were supplemented with 0.50 % agar. The assays were photographed and measured after 48 h of incubation at 28 °C. Abbreviations: wt-p53, wild-type strain 306 with empty vector pUFR053; ∆*hrpB*, *hrpB* mutant; and ∆*hrpB*-p53*hrpB*, complemented *hrpB* mutant. Values are expressed as the means ± standard deviations of three independent experiments. Different letters indicate significantly different p-values (p ≤ 0.05) in Tukey’s tests

RNA helicases have previously been reported to be involved in bacterial motility [12, 30]. The ∆*hrpB* and wild-type strains were both tested to determine their motility on 0.5 % agar SB medium, which is used in sliding analyses in *X. citri*. In *X. citri*, sliding motility was promoted by EPS and inhibited by type IV pili [19, 27]. Deletion of *hrpB* increased cell dispersion in *X. citri* on SB medium plates (*P* <0.05, turkey test) (Fig. 4a). On the plate, the diameters of the growth zones that resulted from migration away from the inoculation points on the agar surface were approximately 1.41 cm for ∆*hrpB* and 0.61 cm for the wild-type strain after 48 h at 28 °C (Fig. 4b). Swimming analyses were also performed, but no difference was observed between the mutant and wild-type strains (data not shown). The complemented strain showed results similar to those of the wild-type strain, indicating that the motility phenotype of the mutant was restored (Fig. 4a and b). Growth curve assays were performed, and no difference was observed between the strains, indicating that the difference observed in the motility assays was not related to growth.

### HrpB is important to *X. citri* survival on host leaves and contributes to virulence

**Fig. 5:**
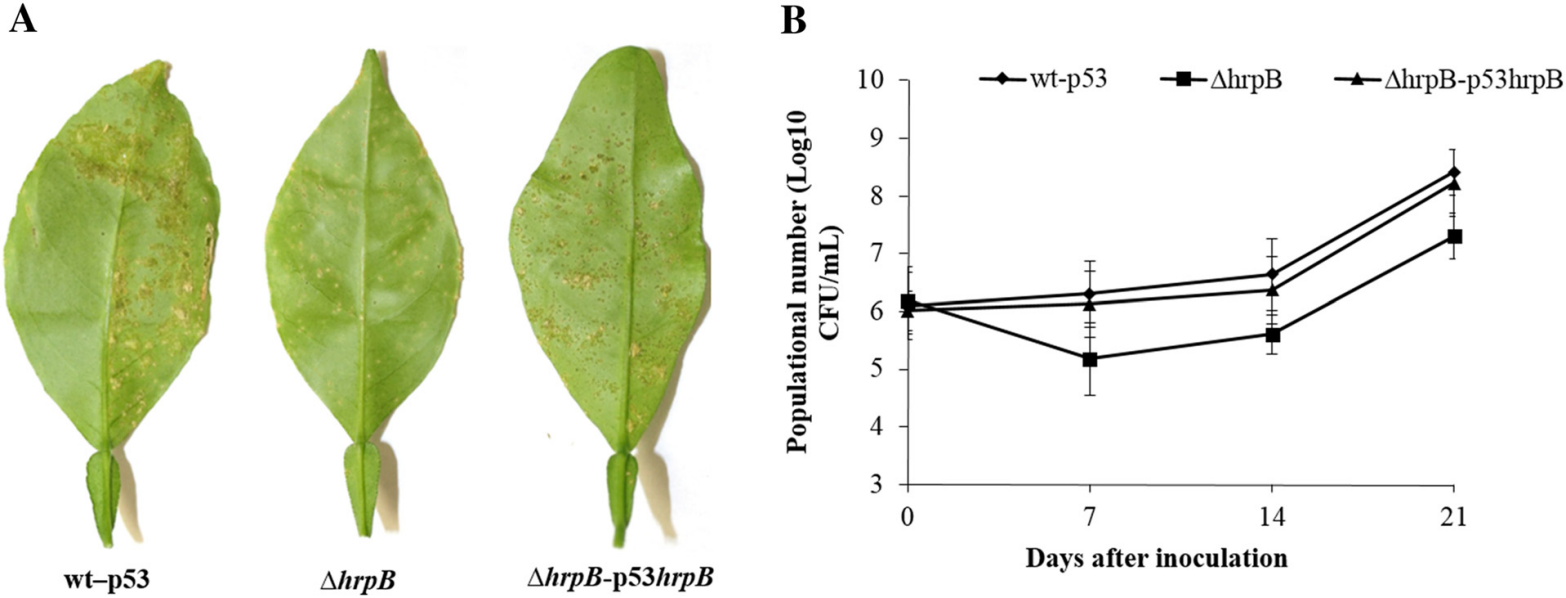
Pathogenicity assay and growth of *X. citri* subsp. *citri* strains in planta. **a** Symptoms were analyzed on the lower the surfaces of sweet orange leaves at 21 days post-inoculation (d.p.i.). **b** *In vivo* bacterial growth in populations of wild-type, ∆*hrpB* and a complementary strain of *Xanthomonas citri* subsp. *citri* (*X. citri*) that were inoculated on the sweet orange leaves by spraying the leaves with bacteria at a concentration of 10^8^ CFU/mL. Bacterial cells were extracted from the leaves at different time points after inoculation. The leaves were homogenized in MgCl_2_ and then plated on appropriate media after serial dilution. Colonies were counted after a 2-day incubation at 28 °C. Abbreviations: wt-p53, wild-type strain 306 with empty vector pUFR053; ∆*hrpB*, *hrpB* mutant; and ∆*hrpB*-p53*hrpB*, complemented *hrpB* mutant. Values are expressed as the means ± standard deviations of three independent experiments

The data from the motility and biofilm formation assays together indicated that there was a reduction in adhesion that could lead to decreased survival in *X. citri* on its sweet orange host. Populations of different strains were quantified at different post-spraying time points on citrus leaf surfaces. At seven days after the initial inoculation, the ∆*hrpB* population was smaller than the population of the wild-type strain 306 (Fig. 5b). Delayed symptoms were observed in the ∆*hrpB* strain compared to the wild-type strain. At 21 days post-inoculation (dpi), the number of canker lesions on leaves infected with ∆*hrpB* was significantly less than the number on leaves inoculated with the wild-type strain (Fig. 5a). Symptoms induced by ∆*hrpB* could be restored to wild-type levels by complementation with plasmid-borne *hrpB* (Fig. 5b). However, there was no difference between the wild-type and the ∆*hrpB* strains in growth or symptom development when they were inoculated into the host leaves via infiltration using a low concentration of bacteria (Additional file 1: Figure S1). These findings suggest that *hrpB* plays an important role in the initial stages of infection in leaves, probably before entry of the pathogen into host plant intercellular spaces.

### HrpB regulates the expression of type IV pili (T4P) genes

**Fig. 6:**
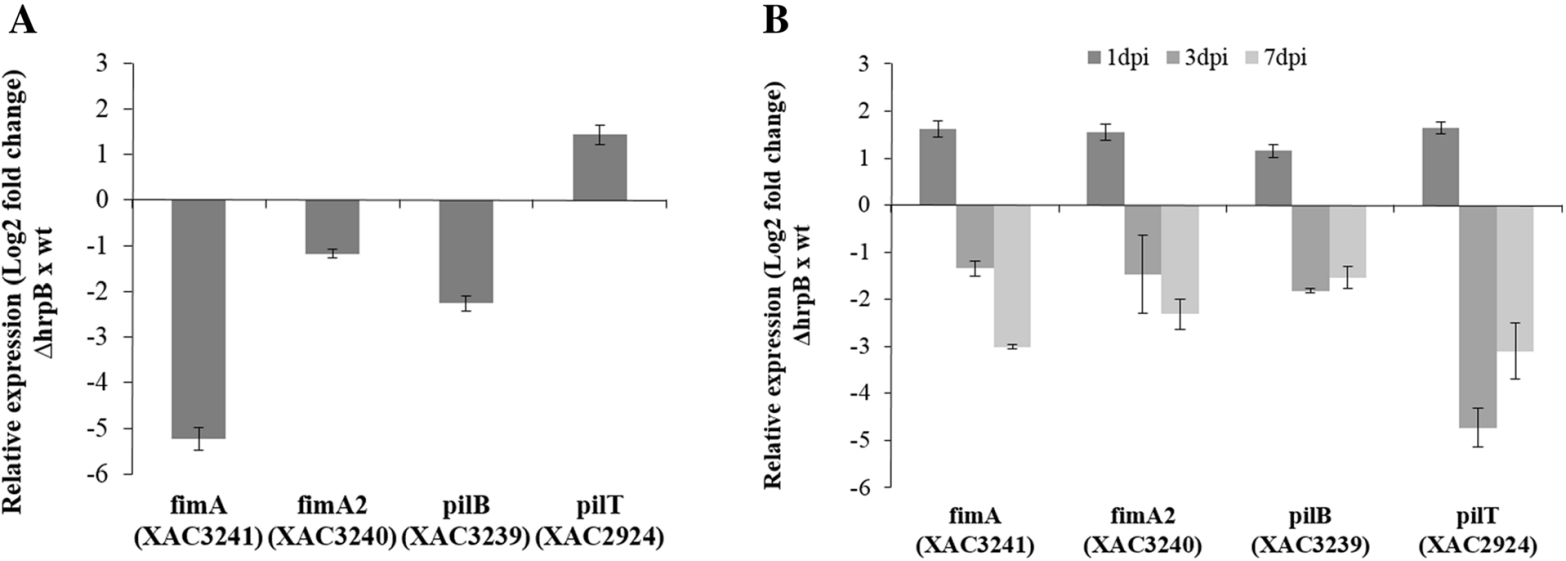
Comparison of type IV pili gene expression (using qRT-PCR) in the wild-type and the ∆*hrpB* bacteria that were cultured in NB medium and *in planta*. **a** Relative expression levels on NB medium. **b** Relative expression levels *in planta*. The data are presented as the ratio (Log_2_-fold change) of the transcript number in ∆*hrpB* compared to the number in wild-type. The DNA gyrase subunit A-encoding gene *gyrA* was used as an endogenous control. Both qRT-PCR assays were repeated twice with similar results, and three independent biological replicates were performed each time

To gain new insights into the roles of *hrpB* in biofilm formation and to explain the reduction observed in adherence in the *hrpB* mutant, we performed qRT-PCR assays using total RNA from wild-type and ∆*hrpB* strains that were grown in NB medium and in orange leaves that were inoculated by spraying. We determined the expression levels of four type IV pili genes (T4P) based on our results and previous studies that showed that mutations in type IV pilus gene are associated with increased motility and decreased biofilms [26, 27]. The genes *fimA*_XAC3241_ and *fimA2*_XAC3240_, which encode for the major pilin subunit [26], and *pilB*_XAC3239,_ an ATPase gene that is required for T4P polymerization [27], may belong to the same operon that exhibited lower expression levels in the ∆*hrpB* strain than in the wild-type strain on NB medium (Fig. 6a). On the other hand, the expression of *pilT*_XAC2924_, an ATPase that is essential for T4P biogenesis [31], was not different from that observed in the wild-type (Fig. 6a). Likewise, qRT-PCR results from an analysis of *X. citri* recovered from leaves showed that there were no changes in the expression of the studied genes between ∆*hrpB* and the wild-type strain at 1 dpi. However, as shown in Fig. 6b, at 3 and 7 dpi, the expression levels of *fimA*, *fimA2* and *pilB* were reduced in the ∆*hrpB* strain compared to the wild-type strain. Analysis of the results from qRT-PCR suggested that the ∆*hrpB* phenotype reflects the impaired expression of *fimA*, *fimA2*, and *pilB*, which encode the essential components of the type IV pili machinery.

### Mapping of the 5′ untranslated region of the *fimA* (XAC3241) transcript

**Fig. 7:**
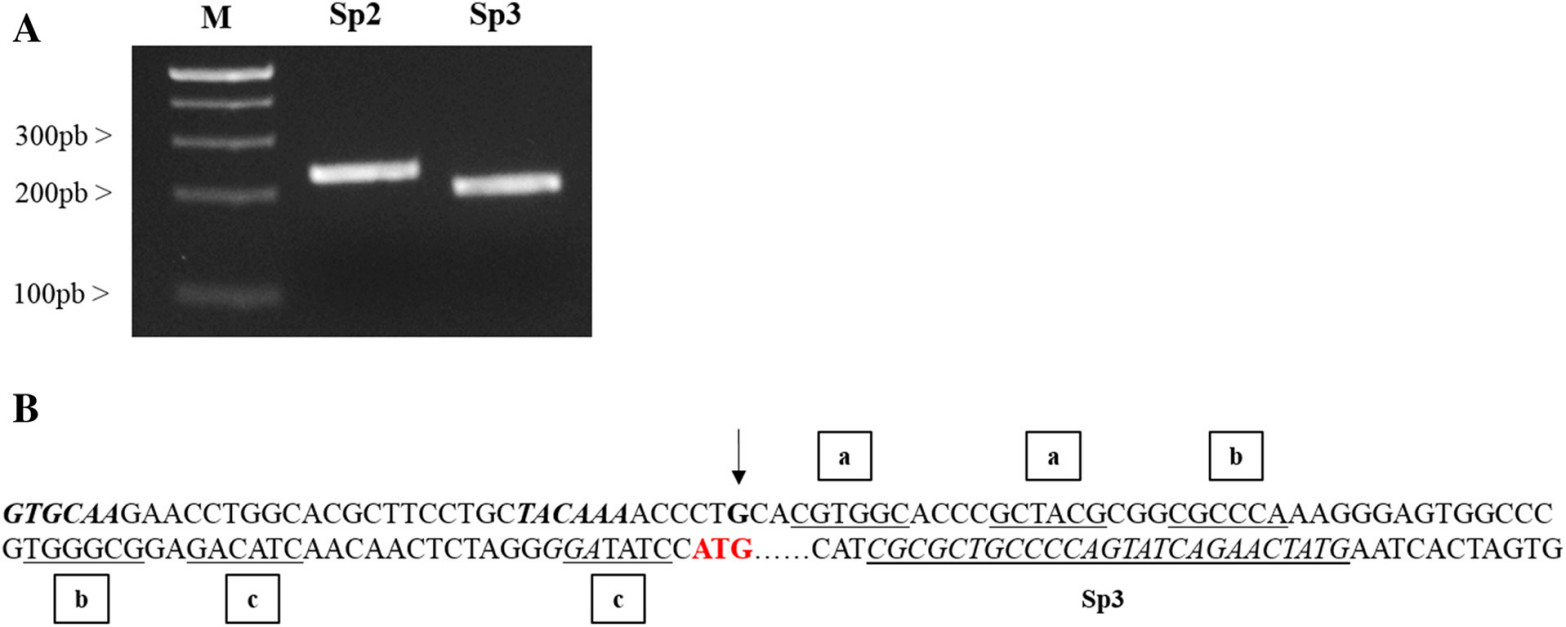
Identification of the *fimA* (XAC3241) transcriptional start site in *X. citri* subsp. *citri*. The transcriptional start site was determined using 5′ RACE. **a** A specific PCR product was detected after amplification was performed using reverse-transcribed cDNA with gene-specific primers for the *pilA* gene and with the adapter primers SP2 and SP1 (Roche). **b** Sequencing of the PCR products showed that the nucleotide indicated by arrow is the transcription start site of *fimA* in *X. citri*. The +1 nucleotide is indicated with an arrow, and the −35 and −10 sequences are shown in bold and italic, respectively. ATG is indicated in red, and the specific primer used to amplify the fragment is shown in bold. The letters represent the hairpins that were identified in the RNA: a = 1^st^ hairpin, b = 2^nd^ hairpin and c = 3^rd^ hairpin

**Fig. 8:**
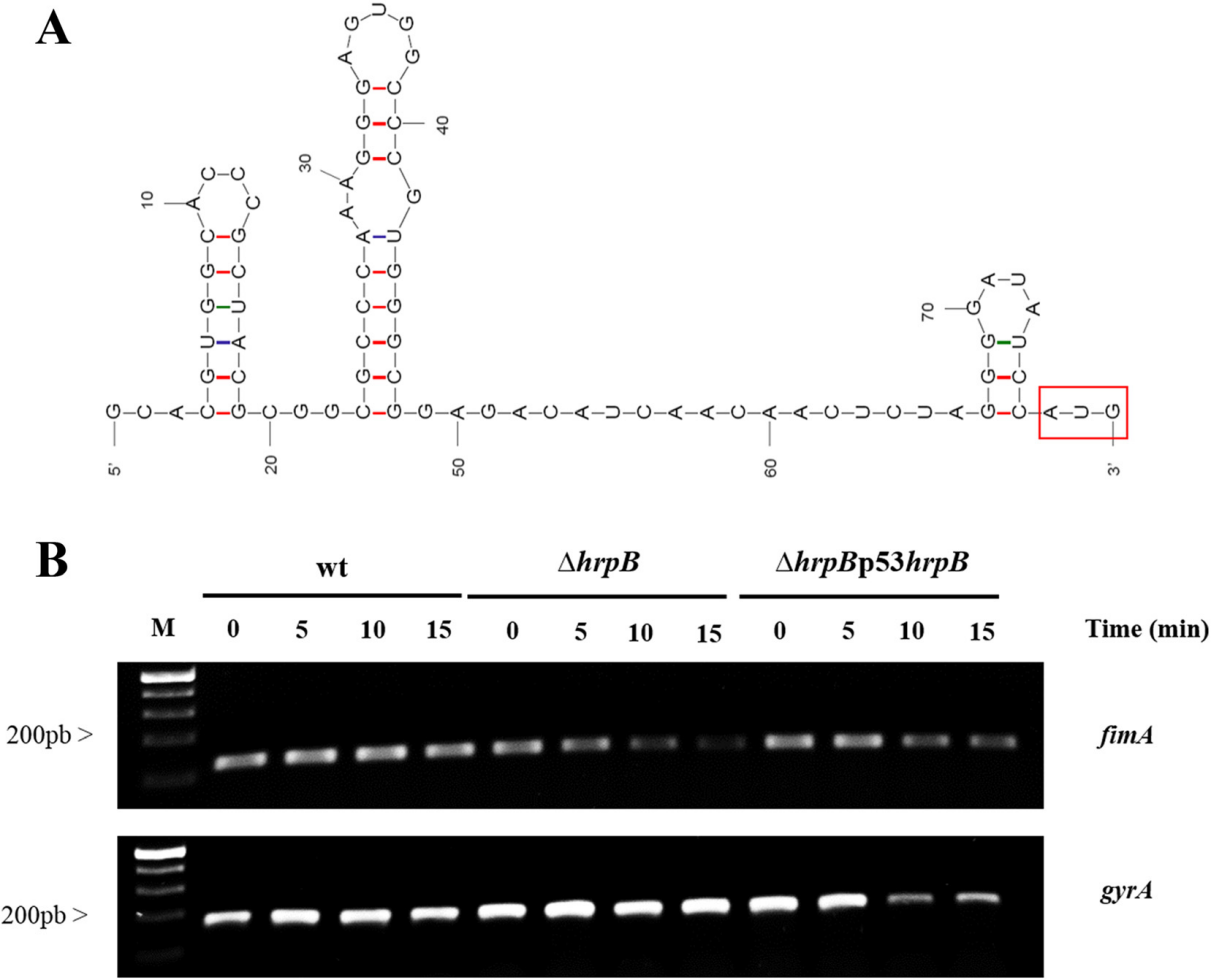
Analysis of *fimA* (XAC3241) mRNA stability. **a** The predicted secondary structure of the *fimA* 5′ untranslated region was obtained using MFold software [32]. The first codon, AUG, is shown in a red box. **b** Analysis of *fimA* mRNA abundance in the wild-type, ∆*hrpB* and complementation (∆*hrpB*-p53*hrpB*) strains was performed using RT-PCR. Analysis of the wild-type, ∆*hrpB* and complementation (∆*hrpB*-p53*hrpB*) strains that were grown in NB medium to OD 600 nm = 0.5 and then treated with 10 mg/mL ciprofloxacin before being harvested at several time points. *fimA* mRNA stability was then determined in the wild-type and ∆*hrpB* strains using RT-PCR. Total RNA was isolated, and 2 mg of RNA was used to synthesize the cDNA that was used for RT-PCR in 25 mL reactions. The reactions were subjected to PCR amplification for 22 cycles. Ten microliters of each reaction was resolved on a 1.5 % agarose gel. The stability of the *fimA* transcript was evaluated, and *gyrA* was used as the control to normalize the *fimA* amplification products

Previous studies have suggested that RNA helicases act by unwinding the secondary structure of the 5′ UTR in the target mRNA, which enables binding and scanning by the small ribosomal subunit to the start codon, AUG [1, 2]. To determine the mechanism by which HrpB acts on the *fimA* transcript, we mapped the 5′-UTR of the *fimA* mRNA to check the presence of putative secondary structures in this region. The 5′ UTR of *fimA* was determined using rapid amplification of 5′-cDNA ends (5′RACE). The RACE PCR product was sequenced to identify the specific transcriptional start site of *fimA*, which is located 113 nucleotides upstream of the first codon (Fig. 7a). Analysis of the 5' RACE results showed that the −35 and −10 regions were the locations for the ribosome binding site and the first codon for *fimA*_XAC3241_ in *X. citri* (Fig. 7b). Mfold [32] was used to predict the RNA structure of this region, and the analysis suggested that the *fimA* 5′ UTR may contain three different loops (Fig. 8a).

### Effects of *hrpB* on *fimA* transcript stability

Because the qRT-PCR analysis showed that there were lower levels of the transcripts of the *fimA*, *fimA2* and *pilB* genes in ∆*hrpB* cells that were grown in rich medium and on plant surfaces, we next analyzed the stability of the *fimA* (XAC3241) transcript in the wild-type and ∆*hrpB* strains. To measure the stability of the *fimA* mRNA in the wild-type and ∆*hrpB* strains, the abundance of the *fimA* transcript was analyzed using the *gyrA* transcript as a control in RT-PCR. Prior to the reactions, transcription was blocked by adding 10 μg/mL ciprofloxacin (Sigma, USA) to the *X. citri* cell cultures. The relative abundance of the *fimA* transcripts was estimated from the specific PCR products that were observed on the agarose gels in comparison to the abundance of the control. The data showed that there was a significant reduction in the abundance of the *fimA* mRNA after 10 and 15 min in ∆*hrpB* compared to the wild-type strain (Fig. 8b). These results revealed that the *fimA* transcript may be more stable in wild-type *X. citri* strains than in the *hrpB* mutant. This reduced stability may contribute to the differences observed in *fimA* transcript levels between the ∆*hrpB* and the wild-type *X. citri* strains in the qRT-PCR experiments (Fig. 6).

## Discussion

Stable adhesion to an appropriate surface is the first step in biofilm formation [33]. The ability of *X. citri* to form biofilms enhances the epiphytic persistence of this species on host leaves, which plays an important role in the early stages of infection [18, 21]. The present study indicates that a putative DEAH-box ATP-dependent RNA helicase, HrpB, may perform many roles in *X. citri* because it is important for adherence to surfaces and motility and for the epiphytic survival of the bacteria on citrus leaves, which results in a reduction in the development of symptoms. The phenotype changes observed in ∆*hrpB* may be caused by the positive regulation of T4P genes, such as *fimA* (XAC3241), by the HrpB protein in wild-type *X. citri*. Our results show that the predicted HrpB protein in *X. citri* exhibits several characteristics that are considered typical for RNA helicases [2, 4]. These include a DEAH-like helicase superfamily domain and the presence of an ATP-binding region (GAGKT) (Fig. 1). Previous studies have suggested that DExD/H-box RNA helicases may be essential to these processes [34]. The observed *hrpB* mutants may be caused by the presence of multiple DEAD-box RNA helicases in *X. citri*, such as Xac0293 and Xac0442. Interestingly, four DEAD-box helicases, SrmB, RhlE, CsdA, and RhlB, are present in *E.coli*, and RhlB, RhlE, and CsdA are interchangeable for certain functions [35].

In plant-bacteria interactions, biofilm formation has been implicated in the virulence of several bacterial pathogens [36], including *X. citri* [18, 21, 37, 38]. Different genes have been found to be important for biofilm formation in *X. citri* [20, 21, 33, 38–40]. However, to the best of our knowledge, *hrpB* (XAC0293) has not been previously reported to play a role in biofilm formation in *Xanthomonas*. In *X. citri*, deletion of *hrpB* resulted in a decrease in cell adhesion and consequently less biofilm compared to a wild-type strain on both abiotic and biotic surfaces (Fig. 3). The ∆*hrpB* mutant also showed a reduction in population on leaf surfaces when spray inoculated and a delay in canker development (Fig. 5). Similar phenotypes were also observed in other *X. citri* mutants, including type 4 pilus mutants [18, 41]. As expected, no difference was observed in symptoms when the *hrpB* mutant cells infiltrated into the host the leaves (Additional file 1: Figure S1). Similar results were previously reported in *X. citri* T4P mutants, indicating that T4P plays an important role in the adherence and epiphytic stages of canker disease but not in the mesophyll stage, whereas other mechanisms, such as the type III secretion system, have been shown to be involved [26]. The reduction in adhesion was also observed as a significant increase in sliding motility on a semisolid medium (Fig. 4). The faster movement of the cells may be attributed to a lack of cell-to-cell aggregation. A similar phenotype was observed in *X. citri* when the hemagglutinin-like adhesins that are involved in cell-to-cell aggregation were mutated [33]. In addition, it has been shown that in *X. citri*, sliding motility is inhibited by surface structures, such as T4P, possibly as a result of the increased interactions between the bacterial cells and the substrate [19, 21, 27, 41]. Therefore, *fimA* (XAC3241) and *pilB* (XAC3239) mutants showed increased sliding motility and reduced biofilm formation [26, 27]. Taken together, these results are in agreement with our data, in which we verified that *hrpB* mutation resulted in the repression of TP4 genes, a reduction in adhesion and an increase in movement (Fig. 6).

RNA helicases participate in many aspects of RNA metabolism and have been shown to be involved in different phenotypes, including phenolic acid metabolism [42], cold adaption [10], auto-aggregation [11], motility [30] and biofilm formation [43]. One of the main functions of DEAD/DEAH-box RNA helicases is the binding and remodeling of the secondary structures of RNA molecules. Our results show that the *fimA* 5′ UTR contains three different loops in its structure. These loops could impair the initiation of translation in *X. citri* cells (Fig. 7). Studies of mRNA regulation have shown that mRNA secondary structures in the 5′ UTR can dramatically influence the initiation of translation [44]. Thus, the interaction between HrpB and *fimA* leader sequences may function by unwinding the secondary structure in the 5′ UTR of the mRNA to enable binding and scanning by the small ribosomal subunit to the start codon, AUG, resulting in an increase in the translation efficiency of this mRNA in *X. citri* cells. It has been shown in *Bacillus subtilis* that two cold-induced putative DEAD-box RNA helicases, CshA and CshB, destabilize the secondary structures in mRNA that prevent the initiation of translation so that the single-stranded mRNA can be successively bound by cold shock proteins to prevent refolding until translation is initiated at the ribosome [10]. Similarly, in *E. coli*, a DEAH-box RNA helicase that is involved in the processing of the mRNA of a fimbrial operon is required to alter the RNA structure element that is upstream of the processing site, which consequently increases the stability and translation of the fimbrial transcript [13]. These results suggest that the degree to which translation is inhibited in the *hrpB* mutant was correlated with the 5′ UTR secondary structure of the *fimA* mRNA.

Translational repression often leads to the rapid decay of mRNA [45]. When the translation of mRNA is inhibited, transcripts are generally more susceptible to degradation by RNase E [46]. Consistent with these findings, an assay used to assess *fimA* stability revealed that the mRNA of ∆*hrpB* was less stable than that of the wild- type (Fig. 8), which would account for the reduced steady-state levels of the mRNA. These data were then reinforced by our qRT-PCR data. These findings allowed us to speculate about different possibilities that might explain *fimA* mRNA decay observed in the mutant. Our first hypothesis was that HrpB may unwind the loops in the 5′ UTR of the *fimA* mRNA to enable ribosomal binding, which protects the mRNA against decay. Previous studies have indicated that ribosome binding to a ribosome-binding site (RBS) assists in protecting mRNAs from attack by ribonucleases [44, 46]. This notion is supported by studies that have examined the influence of RBS mutations on RNA decay [47]. For example, experiments in both *E. coli* and *B. subtilis* have shown that a variety of mRNAs can be significantly destabilized by mutations in the Shine-Dalgarno element that interfere with ribosome binding by markedly reducing complementarity to 16S rRNA [46, 48]. Conversely, mutations that improve ribosome binding can prolong mRNA longevity [49]. Furthermore, a second possibility that may explain the reduced abundance of transcripts in ∆*hrpB* would be that HrpB is associated with the recruitment of other regulatory factors. In studies of other DExD/H RNA helicases, it has been proposed that when one of these factors binds to a 5′UTR, it may modulate its activity physically to recruit a complex of proteins, which could potentially defend the 5′mRNA against decay [1, 13]. However, to fully understand the role of HrpB in *fimA* mRNA stability, the factors that interact with this putative ATP-dependent RNA helicase need be identified and characterized. The third hypothesis involves the affinity of some ribonucleases to double-stranded RNA. Double-stranded RNAs are targeted by specific ribonucleases, such as RNAse III [49]. If HrpB destabilizes the loops in the 5′UTR of the *fimA* mRNA, the putative ribonuclease would not be able to bind to its target, and HrpB could thereby prevent the degradation of the mRNA.

The ATP-dependent RNA helicase HrpB appears to positively regulate the *fimA* mRNA in addition to other genes in the same operon that are involved in adherence, biofilm formation, motility and the development of citrus canker disease. Further biochemical studies are required to determine the mechanisms involving the HrpB protein in this process. To the best of our knowledge, this is the first time that a DEAH-box RNA helicase has been implicated in the regulation of type IV pili genes in *Xanthomonas* and the first time that a RNA helicase has been shown to be important for motility, biofilm formation and epiphytical survival.

## Conclusions

In this work, we characterized the *hrpB* gene, which encodes an ATP-dependent RNA helicase, in *X. citri.* We demonstrate that the HrpB protein in *X. citri* is involved in biofilm formation, motility and survival on leaf tissues. Quantitative reverse transcription-PCR assays indicated that deletion of *hrpB* reduced the expression of three IV pili genes, including *fimA*. The *fimA* mRNA predicted structure indicated that the *fimA* 5′ UTR may contain three different loops. The ATP-dependent RNA helicase HrpB appears to positively regulate the abundance of the mRNA of *fimA* by promoting the stability of the *fimA* RNA.

## Methods

### Bacterial strains and growth conditions

The bacterial strains and plasmids used in this study are listed in Table 2. *E. coli* DH5α cells were grown at 37 °C in Luria-Bertani (LB) medium (1 (w/v) tryptone, 0.5 (w/v) yeast extract, and 1 % (w/v) sodium chloride, pH 7.5) shaking at 200 rpm or on plates. *X. citri* wild-type (ampicillin-resistant) [25] and mutant strains were grown at 28 °C in nutrient broth (NB; Difco, Detroit, MI) shaking at 200 rpm or on nutrient agar (NA; Difco, Detroit, MI) plates. When required, antibiotics were added at the following concentrations: ampicillin (Ap) 100 μg/mL and gentamycin (Gm) 5 μg/mL.

**Table 2:** Strains and plasmids used in this study

Strains and plasmids	Characteristics	Reference or source
Strains		
*Escherichia coli*		[50]
DH5α	F- *recA*1 * endA*1 *hsdR*17 *supE*44 thi-1 *gyrA*96 real1 ∆ (*argE*-*lacZ*YA) 169 Ø lazA ∆ M15	Promega
*Xanthomonas citri* subsp. *citri*		
306	Syn. *X. axonopodis* pv. *citri* strain 306; wild-type, Rf^r^, Ap^r^	[25, 51]
∆*hrpB* (*hrpB*-)	*hrpB* (XAC0293): pNPTS138 derivative	This study
∆*hrpB*-p53*hrpB* (*hrpB*+)	*hrpB* (XAC0293) contained in pUFR053, Gm^r^	This study
Plasmids		
pGEM T-Easy	PCR cloning and sequencing vector, *lacZ*, Ap^r^	Promega
pNPTS138	Km^r^, *sacB*, *lacZ*α^+^	M. R. Alley, unpublished
pUFR053	IncW Mob^+^ *mob* (P) *lacZ*α^+^ Par+, Cm^r^, Gm^r^, Km^r^, shuttle vector	[52]
pNPTS_*hrpB*	*hrpB* (XAC0293) gene cloning on pNPTS138	This study
p53_*hrpB*	*hrpB* (XAC0293) gene cloning on pUFR053	This study

Ap^r^, Cm^r^, Gm^r^, Km^r^, and Rf^r^ indicate resistance to ampicillin, chloromycetin, gentamicin, kanamycin and rifamycin, respectively

### DNA manipulations

Bacterial genomic DNA and plasmid DNA were extracted using a Wizard genomic DNA purification kit and a Wizard miniprep DNA purification system according to the manufacturer’s instructions (Promega, Madison, WI, USA). The concentration and purity of the DNA were determined using a Nanodrop ND-1000 spectrophotometer (NanoDrop Technologies, Wilmington, DE, USA). PCR was performed using standard procedures [53] with Pfu DNA polymerase (Promega Corporation, Madison, WI). The restriction digestions and DNA ligations were performed according to the manufacturer’s instructions (New England Biolabs, USA).

### Construction of the *hrpB* deletion mutant and the complemented strain of *X. citri*

To construct the *hrpB* deletion mutant, approximately 1 kb of the upstream and downstream regions of the *hrpB* gene (XAC0293) was amplified using PCR from genomic DNA obtained from *X. citri* strain 306 using the following primer pairs: 0293mF1Hind (5′ CGTGTTCACCGAGGACAGTGGCCTG 3′) and 0293mR1Bam (5′ ATAAGGATCCAAAAGCGGGGTCGGTCATGC 3′); and 0293F2Bam (5′ CTATGGATCCGGCACCTGAAACACATGGACC 3′) and 0293mR2Hind (5′ GAATAAGCTTTGCTGCCGGTGGTGGATTGTG 3′), respectively. The PCR products were digested with *Bam*H1, and both fragments were ligated to produce a deletion construct. The resulting fragment was cloned into the pNPTS138 suicide vector (M. R. Alley, unpublished) to generate pNPTS_*hrpB* (Table 2) using the restriction enzymes *Eco*R1 and *Hin*dIII. The plasmids were introduced into *E. coli* by heat-shock at 42 °C according to standard procedures [53] and into *X. citri* by electroporation [54]. The wild-type copy was replaced with the deleted version after two recombination events as previously described [55]. All of the obtained clones were confirmed using PCR.

To complement the *hrpB* knockout mutant, a 2800bp DNA fragment containing the entire *hrpB* gene plus approximately 300 bp of neighboring region was amplified by PCR using total DNA obtained from the *X. citri* wild-type strain 306 as the template and the specific primer pair 0293_p53_F (5′AGGAAAAACATATGGGTACCTTTCCGATCTCCCCGTTATTGCC 3′) and 0293_p53_R (5′AGGAAGGATCCTGCGGTACCGGTGCCACGTGGTTTTGCTCTGT 3′). The amplified DNA fragment was cloned into pUFR053 [52] at the *Kpn*I restriction site to obtain the recombinant plasmid p53_hrpB (Table 2), which was used for genetic complementation. The construction was confirmed using sequencing. The recombinant plasmid p53_*hrpB* was transferred into ∆*hrpB* using electroporation, and cells were selected on NA using gentamicin, resulting in the strain ∆*hrpB*-p53*hrpB* (*hrpB*+) (Table 2).

### Biofilm formation assays

Biofilms that formed on polystyrene and glass surfaces were examined as previously described [56], with modifications. After 24, 48 and 72 h of incubation, optical density was measured at 590 nm, and the data were normalized at an OD of 600 nm. Quantitative measurements of 24 replicates were performed for each *X. citri* evaluated strain. Assays of biofilm formation on leaf surfaces were performed as previously described [22]. Briefly, a 20 μL volume of each bacterial suspension (10^8^ CFU/mL) was incubated on the abaxial surface of Valencia sweet orange leaves, which were maintained at 28 °C in a humidified chamber. At 24, 48 and 72 h after the start of incubation, biofilm formation was visualized on the leaf surfaces using crystal violet staining. Leaf discs from staining spots were excised, dissolved in 1 mL ethanol:acetone (70:30, v/v) and quantified by measuring the optical density at 590 nm. Data from both experiments were statistically analyzed using one-way analysis of variance (ANOVA) (*p* < 0.05), and values are expressed as the means ± standard deviations.

### Motility assays

To test cell motility, bacteria were grown overnight in NB medium. A 3 μL volume of bacterial cultures with OD 600 = 0.3 was then spotted onto the surface of a plate containing SB medium plus 0.5 % (wt/vol) agar (Difco, Franklin Lakes, NJ) [27] for the sliding motility tests or NYGB medium 0.25 % (wt/vol) agar [19] for the swimming motility tests. Plates were incubated at 28 °C for 48 h. The diameters of the circular halos that were occupied by the strains were measured, and the resulting values were taken to indicate the motility of *X. citri* strains. The experiments were repeated three times with three replicates each time. The diameter measurements were statistically analyzed using one-way analysis of variance (ANOVA) (*p* < 0.05), and the values are expressed as the means ± standard deviations of three independent experiments.

### Pathogenicity assays

Pathogenicity assays were performed as previously described [39]. Briefly, fully expanded, immature leaves were obtained from young (approximately 10-week-old) sweet orange (*Citrus sinensis* cv. Valencia) plants that were prepared in a quarantined greenhouse at the Citrus Research and Education Center (Lake Alfred, FL). *X. citri* strains (*X. citri* 306 wild-type, Δ*hrpB* and ∆*hrpB*-p53*hrpB*) that were grown in selective antibiotic NB medium overnight at 28 °C were centrifuged at 4800 rpm and then resuspended in 1 % phosphate buffer (pH 7.0). Bacterial suspensions from each strain were inoculated by pressure infiltration (10^5^ CFU/ml) and spraying (10^8^ CFU/mL). Phosphate buffer was used as the control in non-infected plants. All plant inoculations involved a minimum of three immature leaves from each plant, and three plants were inoculated for each bacterial strain. The plants were kept in a greenhouse at the Citrus Research and Educational Center (Lake Alfred, FL, USA) at a temperature of 28 ± 4 °C in high humidity for 21 days. Disease symptoms were photographed at 7, 14 and 21 days post-inoculation, and both tests were independently repeated two times.

### Epiphytic growth on citrus leaves

To obtain measurements to analyze bacterial epiphytic survival, populations of the pathogen were isolated from the leaves that were inoculated by spraying as described above. At 0, 7, 14 and 21 days post-inoculation, three leaves with similar sizes were randomly collected from three different plants. The leaves were immersed in 10 mL of 1 % phosphate buffer in Falcon flasks (50 mL). Bacterial cells were collected and then vortexed for three minutes to homogenize the tissue. Epiphytic bacterial numbers were determined in serial dilutions of these suspension and then plated on NA medium with the appropriate antibiotics. Colonies were counted after 2 days of incubation at 28 °C. The assays were independently repeated two times with three replicates.

### RNA extraction and quantitative reverse-transcription polymerase chain reaction (qRT-PCR)

To quantitatively analyze gene expression, we used RNA obtained from *X. citri* wild-type and *hrpB* mutant (Δ*hrpB*) strains using two methods: NB medium and the leaves of sweet orange plants that were inoculated by spraying. For the experiments performed using NB medium, the strains were grown in 10 mL of NB at 28 °C while shaking, and both bacterial cultures were collected in the middle exponential stage (OD = 0.8). RNA was immediately stabilized by mixing it with 2 volumes of RNA-protecting bacterial reagent (Qiagen, CA, USA. It was then incubated at room temperature for 5 min. Bacterial cells were centrifuged at 5,000 × g for 10 min, and the cell pellets were then treated with lysozyme. RNA extraction was then performed using an RNeasy minikit (Qiagen, Valencia, CA). For the epiphytical assays, 3 different plants were inoculated with each strain at 10^8^ CFU/mL. At different timepoints after infection (1, 3 and 7 days after inoculation), three leaves were collected from each plant to perform RNA extraction using a RNase plant mini kit (Qiagen, CA, USA). Contaminated genomic DNA was removed from the RNA during both experiments by processing the preparation using a TURBO DNA-free kit (Ambion, TX, USA), and RNA purity and quantity was determined using a ND-8000 Nanodrop spectrophotometer (NanoDrop Technologies, U.S.A.). For qRT-PCR assays, 4 genes in the T4P system (XAC3241, XAC3240, XAC3239 and XAC2924) were chosen for gene expression analyses using the primers shown in Table 3. Reverse transcription was performed using an iScript cDNA Synthesis kit (Bio-Rad) according to the manufacturer’s protocol. Quantitative amplification of the resulting cDNA (1 μg) was performed using 0.3 mM of each primer (Table 3) and SYBR Green/ROX qPCR Master Mix (Qiagen) according to the kit instructions in an ABI7300 Real-Time System (Applied Biosystems). Relative expression was evaluated using the 2^–∆∆CT^ method. *gyrA* was used as the endogenous control. Both types of quantitative real-time PCR experiments were performed using three biological replicates.

**Table 3:** Primers used for qRT-PCR

Genes	Sequences
*gyrA* (XAC1631)	F, GCCTACATTTTGACGGCCAC
	R, ACCGACGAAGTGCTGTTGAT
*fimA* (XAC3241)	F, GAAGCAACAGGGTTTCACGC
	R, TATGTTGCCAAGTCGCAGGT
*fimA2* (XAC3240)	F, TAGCAGTCGCAGTCAAACCA
	R, TTGCGGGATCGCTATGGAAG
*pilB* (XAC3239)	F, ATTGCTGGCCGAAGGATTCA
	R, TAATGCCGATGACCGACGAG
*pilT* (XAC2924)	F, GAATTCCTCGTAATCGCGCC
	R, CAGGTCCGATGCCTTGTTCT

### 5′ - RACE

The transcriptional start site of *fimA* (XAC3241) was determined using a 3′/5′ RACE Kit (Roche) according to the manufacturer’s instructions. Briefly, total RNA was obtained from *X. citri* wild-type cell cultures that were grown in NB medium to an OD 600 of 1.0. After treating the RNA using a TURBO DNA-free kit (Ambion, TX, USA), the RNA was reverse-transcribed using a gene-specific primer (SP1, Table 4) and then purified before a poly(dA) tail was added to its 3′ end in a reaction with a terminal transferase enzyme. The resulting cDNA was amplified using PCR with the poly-dT primer that was provided in the kit, which anneals at the poly(dA) tail, and a gene-specific primer (SP2, Table 4) that was complementary to a region upstream of the original cDNA primer. The amplicons obtained from the first PCR were submitted to a second-round PCR reaction using the poly dT primer and a distinct gene-specific nested primer (SP3, Table 4) that was internal to the first primer. The PCR products were ligated into the pGEM-T vector (Promega), and three distinct clones were sequenced.

**Table 4:** Primers used for 5′- RACE

Primers	Sequences
SP1	CATAGTTCTGATACTGGGGCAGC
SP2	GGCCAGACCAGCAGTGACCTG
SP3	TCGTATTGCGTTTTACCCGG

### mRNA stability assay

Bacterial cultures of *X. citri* subsp. *citri* wild-type, ∆*hrpB* and the complementation strain (Δ*hrpB-*p53*hrpB)* were grown at 28 °C in NB medium to an OD 600 nm of 0.5 and then treated with ciprofloxacin at a final concentration of 10 mg/ml to inhibit transcription. Samples were collected at 0, 5, 10 and 15 min after treatment with ciprofloxacin. The cells were harvested by centrifugation at 5,000 rpm and used immediately to extract RNA using a RNeasy Mini kit (Qiagen, CA, U.S.A.). Total RNA samples were treated with Turbo RNase-free DNase (Ambion) and quantified using a Nanodrop. A total of 2 μg of treated RNA was used for reverse transcription using an iScript cDNA Synthesis kit (Bio-Rad) according to the manufacturer’s protocol. Reactions were subjected to PCR amplification for 22 cycles using 0.3 mM of each of two primers, *fimA*F (5′ GAAGCAACAGGGTTTCACGC 3′) and *fimA*R (5′TATGTTGCCAAGTCGCAGG 3′), and *Taq* 2x Master Mix (Biolabs). Ten microliters of each reaction was resolved in a 1.5 % agarose gel. Analyses of *gyrA* were performed and used as the controls for normalizing the *fimA* amplified products.

## Additional file

Additional file 1: Figure S1. Pathogenicity assay of *X. citri* subsp. *citri* strains in planta. Symptoms were analyzed on the lower the surfaces of sweet orange leaves at 21 days post-inoculation (d.p.i.) of wild-type, ∆*hrpB* and a complementary strain of *X. citri* that were inoculated on the sweet orange leaves by infiltration the leaves with bacteria at a concentration of 10^5^ CFU/mL. Abbreviations: wt-p53, wild-type strain 306 with empty vector pUFR053; ∆*hrpB*, *hrpB* mutant; and ∆*hrpB*-p53*hrpB*, complemented *hrpB* mutant. (PNG 1241 kb)
