# Supplementary figures and images for: The ATP-dependent RNA helicase HrpB plays an important role in motility and biofilm formation in Xanthomonas citri subsp. citri
Source: BMC Microbiol. 2016 Mar 23;16:55. doi: 10.1186/s12866-016-0655-1 (PMC4804567; doi:10.1186/s12866-016-0655-1)

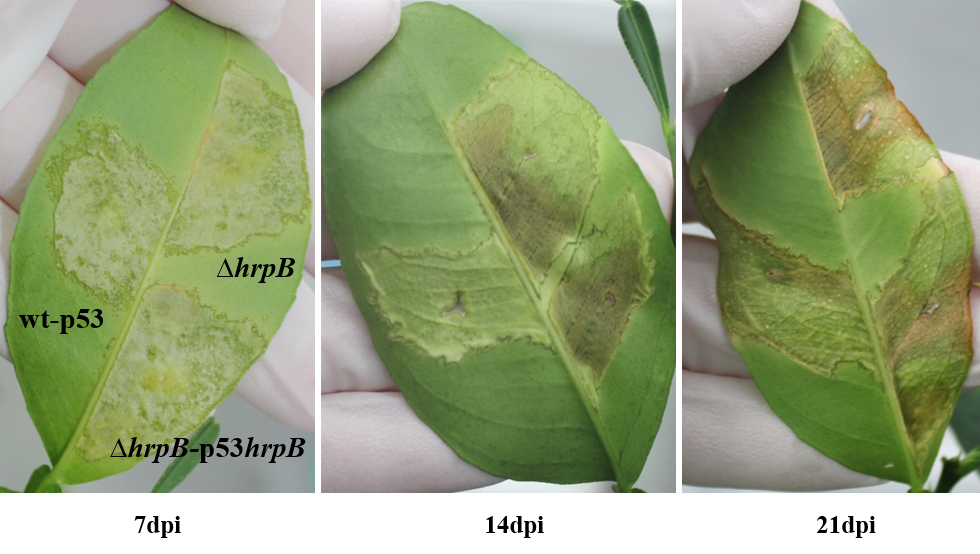

Supplement: Additional file 1: Figure S1. — Pathogenicity assay of X. citri subsp. citri strains in planta. Symptoms were analyzed on the lower the surfaces of sweet orange leaves at 21 days post-inoculation (d.p.i.) of wild-type, ∆hrpB and a complementary strain of X. citri that were inoculated on the sweet orange leaves by infiltration the leaves with bacteria at a concentration of 105 CFU/mL. Abbreviations: wt-p53, wild-type strain 306 with empty vector pUFR053; ∆hrpB, hrpB mutant; and ∆hrpB-p53hrpB, complemented hrpB mutant. (PNG 1241 kb) [file 12866_2016_655_MOESM1_ESM.png]
